# It is all about timing: decompressive hemicraniectomy for malignant middle-cerebral-artery infarction

**DOI:** 10.1055/s-0043-1768674

**Published:** 2023-05-09

**Authors:** Kosmas Macha, Stefan Schwab

**Affiliations:** 1 Friedrich-Alexander-University of Erlangen-Nuremberg, University Hospital Erlangen, Department of Neurology, Erlangen, Germany.

Decompressive hemicraniectomy (DHC) is the most effective intervention to treat the space-occupying effect of malignant cerebral infarction. Therefore, it is recommended by international guidelines to improve survival and potentially neurological outcome in selected patients with malignant middle-cerebral-artery (MCA) infarction. 1 In several studies, timing was identified as a relevant factor for the treatment effect of hemicraniectomy in patients with malignant cerebral infarction. 2 3 Later, the randomized HAMLET trial 4 confirmed this finding, showing reduction of case fatality and poor outcome in patients treated within 48 hours of stroke onset but no effect on functional outcome in patients with treatment delayed up to 96 hours. Consequently, the pooled analysis of the randomized trials DECIMAL, 5 DESTINY, 6 and HAMLET 4 included only patients treated within 48 hours after stroke onset regardless of symptoms suggesting transtentorial herniation. In this large analysis of randomized patients ( *n* = 93), all < 60 years old with severe stroke (NIHSS > 16), decompressive surgery led to higher rates of survival with mRS ≤ 4 (75 versus 24%; pooled absolute risk reduction 51%; 95% confidence interval [CI]: 34–69)), survival with mRS ≤ 3 (43 versus 21%; 23% [5–41]) and survival irrespective of functional outcome (78 versus 29%; 50% [33–67]). These results translate into impressive numbers needed to treat of 2 for survival with mRS ≤ 4, 4 for survival with mRS ≤ 3 and 2 for survival irrespective of functional outcome. 7 The DESTINY II trial revealed effectiveness of decompressive hemicraniectomy in patients > 60 years old for survival with mRS ≤ 4, indicating nonsevere disability (38 versus 18%; OR 2.91 [95%CI: 1.06–7.49]; *p* = 0.04). 8

In this issue of *Arquivos de Neuro-Psiquiatria* , Rodrigues et al. 9 present a study of 43 malignant MCA stroke patients treated with decompressive hemicraniectomy in a Brazilian tertiary hospital. The authors investigated the time course of hemispheric cerebral volume after decompressive hemicraniectomy using hemispheric volumetric measurements of all computed tomography scans (CT scans) performed during inpatient stay before and after DHC. In the study cohort, decompressive hemicraniectomy was performed 41.88 (Standard deviation 29.32) hours after stroke onset. The peak of the hemisphere volume was reached at day 7 (168.84 [95%CI: 142.08–195.59] hours) after the ischemic event. However, the steepest increase in hemisphere volume was demonstrated in the early phase after stroke onset and ∼ 28% of patients showed ipsilateral mydriasis before DHC.

This highlights the importance of timely selection of malignant cerebral infarction patients requiring and potentially benefiting from decompressive hemicraniectomy.

Advanced cerebral imaging could be supportive for this selection process. In an analysis of the DESTINY registry including 140 malignant middle-cerebral-artery infarction patients treated with decompressive hemicraniectomy and available semiautomatic quantification of infarction, an association of infarct volume with outcome could be demonstrated. In multivariable logistic regression, beside age and stroke severity, infarct size before hemicraniectomy was an independent predictor of unfavorable outcome (OR 1.27 for 10 ml increase [95%CI: 1.12–1.44]; *p* < 0.001). Additionally, the authors calculated an infarction volume threshold for unfavorable outcome with high specificity (94% for the overall cohort and 92% in younger patients [≤ 60 years old]) as > 258 ml before hemicraniectomy. 10

Furthermore, beside the proper selection of malignant cerebral infarction patients with potential to benefit from decompressive hemicraniectomy, quality of the neuro-surgical procedure is crucial for the treatment effect. In an interdisciplinary neurological-neurosurgical study including 60 malignant MCA infarction patients treated with DHC, the incidence of hemicraniectomy-associated parenchymal hemorrhages and hemicraniectomy-associated infarcts was 41.6 and 28.4%, respectively. 11 Small operative bone defects were associated with hemicraniectomy-related bleeding, leading to a significantly increased risk for mortality in these patients (survival rate 55 versus 80%; *p* < 0.05). The authors therefore suggested a bone deficit of at least 12 cm and additional duraplasty, as suboptimal hemicraniectomy might reduce the positive treatment effect and adversely affect functional outcome and mortality.

The late occurrence of the peak hemisphere volume in the study by Rodriguez et al. points at the importance of postsurgical monitoring of the decompressive effect of the hemicraniectomy. Besides neuro-radiological cerebral imaging, bedside examinations by palpation of the hemicraniectomy area, automated pupillometry and midline shift monitoring using (transcranial) ultrasound could play an important role here and should be performed frequently in DHC patients. Here again, timely detection of requirement for and initiation of additional (rescue) intracranial pressure treatments as osmotic therapy or hypothermia in selected patients may be relevant for the patients' clinical course. 12

Even though broader availability of mechanical thrombectomy for large vessel occlusion acute ischemic stroke may reduce the incidence of malignant infarctions, 13 timing is the most important part in the management of patients with incipient malignant MCA- stroke.
